# Supplementary material for: The interactome of intact mitochondria by cross-linking mass spectrometry provides evidence for coexisting respiratory supercomplexes
Source: Mol Cell Proteomics. 2017 Dec 8;17(2):216–32. doi: 10.1074/mcp.RA117.000470 (PMC5795388; doi:10.1074/mcp.RA117.000470)
Supplement: Supplemental Data [file supp_RA117.000470_133922_0_supp_23978_fzffwf.zip › spectra_annotation/mito_spectra_annotation/456-1-15-1-2-1.pdf]

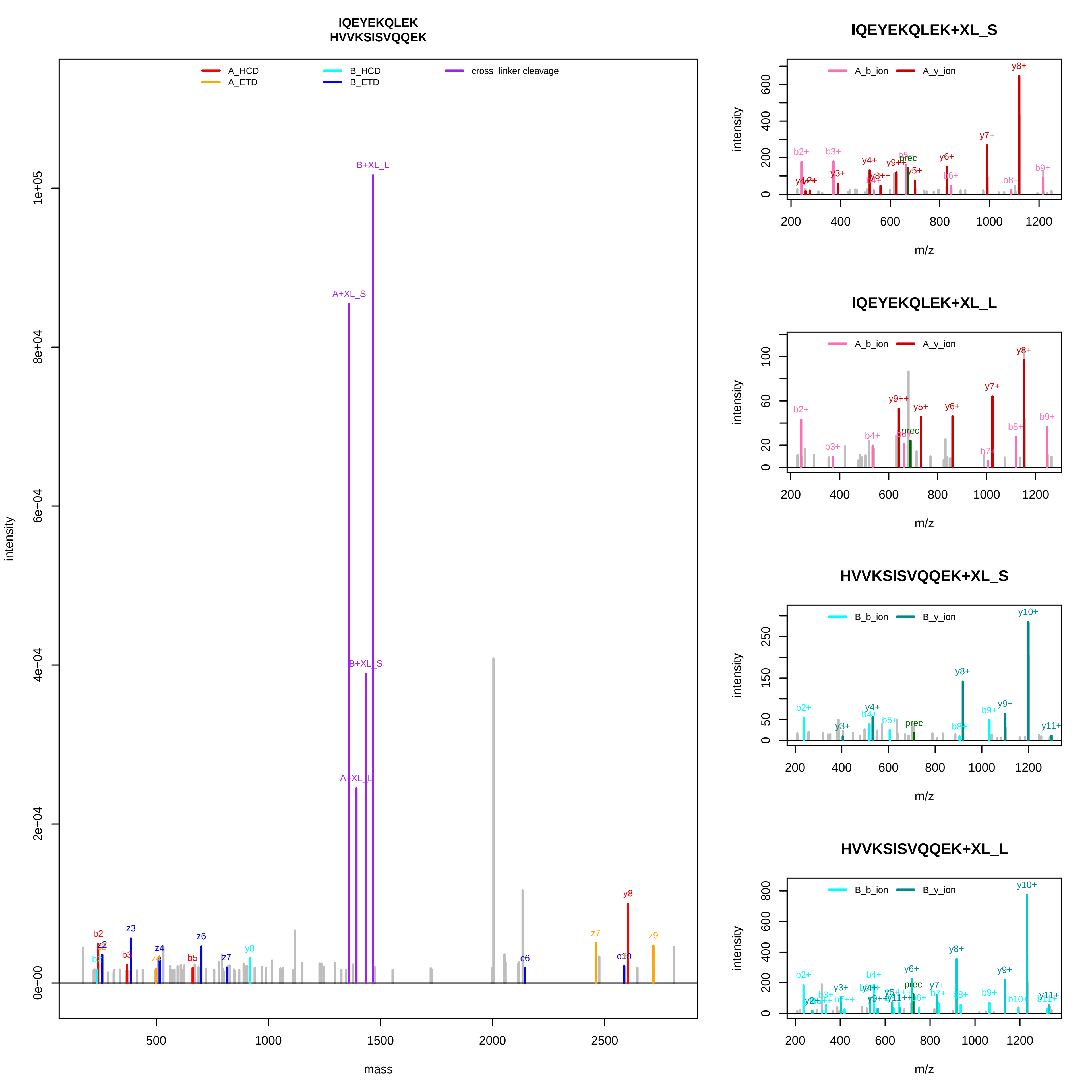

IQEYEKQLEK
HVVKSISVQQEK
A_HCD
A_ETD
B_HCD
B_ETD
cross−linker cleavage
B+XL_L
A+XL_S
B+XL_S
A+XL_L
b2
z2
z3
b3
z4
z6
b5
z7
y8
c6
z7
c10
y8
z9
intensity
mass
0e+00
2e+04
4e+04
6e+04
8e+04
1e+05
500
1000
1500
2000
2500
IQEYEKQLEK+XL_S
A_b_ion
A_y_ion
IQEYEKQLEK+XL_L
HVVKSISVQQEK+XL_S
B_b_ion
B_y_ion
HVVKSISVQQEK+XL_L
m/z
prec
y8+
y7+
y6+
y5+
b9+
b2+
b3+
y10+
y9+
